# Clinical Physicians and Factors Associated With Opening Clinics: An Analysis of National Physician Census Surveys in Japan

**DOI:** 10.7759/cureus.21321

**Published:** 2022-01-17

**Authors:** Masatoshi Ishikawa

**Affiliations:** 1 Takemi Program in International Health, Harvard T.H. Chan School of Public Health, Boston, USA

**Keywords:** national census, longitudinal study, japan, physician, clinic

## Abstract

Background and objective

In Japan, clinics play a key role in providing primary medical care. Data on temporal trends in the number, proportion, and characteristics of clinical physicians, as well as factors associated with the opening of clinics in Japan, have not been adequately explored. This study aimed to analyze the temporal trends as well as factors associated with the opening of clinics in Japan.

Methodology

This study employed a longitudinal study design. Data from the biennial national physician census surveys from 1996 to 2016 were analyzed. It involved all physicians in Japan. As the primary and secondary outcome measures, temporal trends in the number and percentages of physicians were estimated and logistic regression was used to analyze physicians opening clinics.

Results

Recently, the rate of increase in the number of clinical physicians in Japan has been decelerating, and the proportion of older clinical physicians has been increasing. Specifically, the number of clinical physicians increased in the first decade, from 81,888 in 1996 to 95,213 in 2006, but decreased in the second decade to 102,453 in 2016. Apparently, clinical physicians were aging: the number of clinical physicians aged 39 years or younger decreased by 48%, while those aged between 55 and 69 years increased by 91%. The physician-related factors associated with opening clinics include belonging to the age group of 40-54 years, being male, and having an urban area to practice. As of 2016, 39.1% held no board-certified qualifications, whereas 17.1% held board-certified qualifications in areas other than their specialization.

Conclusion

This study based on national surveys revealed that the rate of increase in doctors in Japanese clinics is slowing down and that the proportion of elderly doctors is increasing. The physician-related factors associated with opening clinics include being middle-aged, male gender, and the availability of an urban area for practice.

## Introduction

Medical facilities in Japan consist of hospitals and clinics, or facilities with up to 19 beds. Most clinics do not have beds and each of them is privately operated by an individual physician [1]. Clinics in Japan have comprehensive facilities for consultation and treatment. Certain clinics have facilities and equipment for X-ray imaging, electrocardiography, and blood and urine tests. Such clinics play a key role in providing primary care in Japan [1].

There is no clear distinction between primary care and secondary care in the Japanese healthcare system [2]. In addition, the Japanese healthcare system has historically not adopted the general practitioner system, which functions as a gatekeeper in the UK healthcare system [1]. Moreover, notable characteristics of the Japanese healthcare system are as follows: no local restrictions are placed on opening clinics, and a clinical physician can provide healthcare services in a specialist area even if they do not hold a board-certified qualification in the area [1].

A large number of physicians in Japan undergo resident training at university hospitals. They later change their affiliation to a non-university hospital. Certain physicians choose a career path of opening a private clinic after turning 40 years of age [2]. Previous studies have indicated that the proportion of physicians who leave hospital-based practice for clinic-based practice has remained the same [2]. Motivations for physicians to open their own private clinic are the favorable work environment (it is less demanding than that of hospital-based physicians) [2] and income-related reasons [3,4]. Those who work in a hospital are required to be available for on-call duty and seeing outpatients and inpatients during nighttime hours and on weekends. In addition, surgeons who open a private clinic change their specialization from surgery to internal medicine [5].

It has been observed that the total number of physicians in Japan has been increasing. Therefore, it had been estimated that the number of clinical physicians would increase by 38% from 2004 to 2016 if the proportion of physicians who left hospital-based practice for clinic-based practice remained unchanged [6]. However, according to a biennial survey of physicians, dentists, and pharmacists by the Ministry of Health, Labour and Welfare (MHLW) [7], the number of clinical physicians increased only by 10% from 2004 to 2016.

An examination of changes in the number of clinical physicians in recent years would provide important suggestions for the improvement of primary care in Japan going forward. Data on temporal trends in the number, proportion, and characteristics of clinical physicians, as well as factors associated with the opening of clinics in Japan, are scarce. Because this information is crucial for the planning of healthcare resource allocation, in this study, I aimed to collect and analyze this data to ensure optimal healthcare planning.

Based on the biennial national physician census surveys conducted by the MHLW from 1996 to 2016, the objectives of the present study were to evaluate recent changes in the number and characteristics of physicians working in clinics in Japan and identify the physician-related factors associated with opening clinics.

## Materials and methods

For the survey of physicians, dentists, and pharmacists as per the Medical Practitioners’ Act, all physicians are required to report their status every two years, and the response rate is estimated at approximately 90% [8]. The original survey is conducted every two years for all doctors by the questionnaire method, and the questions include those related to name, registration number, gender, age, years of experience, workplace, facility type, and area of specialty.

I obtained approval from the MHLW to use the individual data from its survey for this research, as per the legal requirements imposed by Statistical Act (H30-0508-3). The means for obtaining the data was provided by the MHLW after anonymizing the data. The study was approved by the Institutional Review Board at the Harvard T. H. Chan School of Public Health (No. 18-1422). The need for informed consent was waived because the survey was mandatory. For each physician in this study, data on the following were evaluated: registration number, gender, age, years of experience, workplace or facility type (municipality and medical institution type), and area of specialty. The clinical physicians were identified by the facility type indicated in their responses. Clinical physicians were defined as those who described themselves as (1) clinic owners or (2) staff. As a result, the number of clinical physicians identified in 1996 was 81,888 (34.1% of all physicians), and it was 102,453 (32.1%) in 2016.

To differentiate the clinical physicians geographically, 344 Secondary Medical Areas (SMAs) were identified and used for this study. The municipality borders that changed owing to mergers were adjusted based on the borders in 2016. The SMAs were subsequently classified into three categories based on the combination of population size and population density in 2016: Group 1 was urban, Group 2 was intermediate, and Group 3 was rural. In Japan, there are no rural criteria comparable to the standards of the US Office of Management and Budget [9]; therefore, the classifications used were based on the MHLW classification position statements regarding the demand for physicians [10].

The number of physicians per 100,000 people in each group of SMAs was calculated using the data for the total number of physicians and the total population taken from the National Census [11]. To account for the differences between the years of the physician data (1996, 2006, and 2016) and the years of the population data (1995, 2005, and 2015), I applied the 1996 physician data to the 1995 population data, the 2006 physician data to the 2005 population data, and the 2016 physician data to the 2015 population data.

To assess the relationship between specialty types and practice locations, physicians who registered their specialties as internal medicine, general surgery, or pediatrics were classified as primary care physicians. As the Japanese health system does not have a formally recognized specialty of primary care comparable to family medicine in the US, internists, general surgeons, and pediatricians play a substantial role in providing primary care [12].

To analyze the data, I first described the demographic and professional characteristics of the clinical physicians in 1996, 2006, and 2016. The reason that 2006 data were analyzed was to compare the data before and after the introduction of the postgraduate mandatory training system. Physicians who graduated in 2004 advanced to a mandatory two-year postgraduate training and became clinicians or physicians scientists in 2006. Therefore, since 2004 was a transition period, 2006 was set as a watershed year.

To identify the factors associated with being clinical physicians in 1998, 2008, and 2016, a multivariable logistic regression analysis was conducted among respondents who were not clinic owners in 1996, 2006, and 2014. Being a clinic owner after two years was deemed a dependent variable, and gender, age, workplace (geographical area and institution type), and primary care specialty were designated as independent variables. Development of cohort data: for example, doctors who responded in both the 1996 and 1998 surveys were linked using the physicians' registration number and identified as the 1996-1998 cohort. In addition, I verified the relationships between types of specialties and specialty certificates held by clinical physicians as of 2016.

The STATA 15.1 software (StataCorp LLC, College Station, TX) was used for all statistical analyses. P-values of less than 0.05 were considered statistically significant.

Patient and public involvement

It was not deemed appropriate or possible to involve patients or the public in the design, conduct, reporting, or dissemination plans of this research.

## Results

Table 1 illustrates the distribution and characteristics of clinical physicians in Japan in 1996, 2006, and 2016.

**Table 1:** Demographic and professional characteristics of clinical physicians in 1996, 2006, and 2016

	1996 Survey		2006 Survey		2016 Survey		1996 to 2016
Total number of subjects	81,888		95,213		102,453		25.1%
% of all physicians	34.1%		34.3%		32.1%		-5.9%
Sex, n, %							
Male	71,185	86.9%	80,468	84.5%	83,067	81.1%	16.7%
Female	10,703	13.1%	14,745	15.5%	19,386	18.9%	81.1%
Age, n, %							
≤39 years	7,478	9.1%	7,251	7.6%	4,721	4.6%	-36.9%
40–54 years	27,348	33.4%	38,014	39.9%	32,884	32.1%	20.2%
55–69 years	27,435	33.5%	28,200	29.6%	45,984	44.9%	67.6%
≥70 years	19,627	24.0%	21,748	22.8%	18,864	18.4%	-3.9%
Years of experience, n, %							
0–14 years	9,404	11.5%	8,322	8.7%	5,476	5.3%	-41.8%
15–29 years	27,551	33.6%	40,160	42.2%	35,077	34.2%	27.3%
30–44 years	23,387	28.6%	26,872	28.2%	42,551	41.5%	81.9%
≥45 years	21,546	26.3%	19,859	20.9%	19,349	18.9%	-10.2%
Position, n, %							
Owner	66,329	81.0%	71,192	74.8%	71,887	70.2%	8.4%
Other physicians	15,559	19.0%	24,021	25.2%	30,566	29.8%	96.5%
Workplace, n, %							
Urban	37,333	45.6%	44,536	46.8%	50,142	48.9%	34.3%
Intermediate	37,554	45.9%	43,323	45.5%	45,310	44.2%	20.7%
Rural	7,001	8.5%	7,354	7.7%	7,001	6.8%	0.0%
Specialty, n, %							
Primary care	47,588	58.1%	50,904	53.5%	51,159	49.9%	7.5%
Others	34,300	41.9%	44,309	46.5%	51,294	50.1%	49.5%
Specialty, n, %							
Internal medicine	35,807	43.7%	39,374	41.4%	40,586	39.6%	13.3%
Ophthalmology	6,028	7.4%	7,573	8.0%	8,395	8.2%	39.3%
Orthopedics	4,946	6.0%	7,017	7.4%	7,796	7.6%	57.6%
Pediatrics	5,844	7.1%	6,472	6.8%	6,581	6.4%	12.6%
Otolaryngology	4,771	5.8%	5,265	5.5%	5,433	5.3%	13.9%
Dermatology	3,557	4.3%	4,587	4.8%	5,411	5.3%	52.1%
Obstetrics and gynecology	5,413	6.6%	5,403	5.7%	5,342	5.2%	-1.3%
General surgery	5,937	7.3%	5,058	5.3%	3,992	3.9%	-32.8%
Psychiatry	1,271	1.6%	2,496	2.6%	3,862	3.8%	203.9%
Gastroenterology	2,718	3.3%	3,275	3.4%	3,389	3.3%	24.7%
Cardiology	965	1.2%	1,471	1.5%	1,967	1.9%	103.8%
Urology	915	1.1%	1,560	1.6%	1,908	1.9%	108.5%
Unknown	875	1.1%	938	1.0%	1,264	1.2%	44.5%
Neurosurgery	387	0.5%	864	0.9%	1,128	1.1%	191.5%
Other	768	0.9%	607	0.6%	1,061	1.0%	38.2%
Psychosomatic internal medicine	74	0.1%	514	0.5%	646	0.6%	773.0%
Pulmonology	255	0.3%	351	0.4%	580	0.6%	127.5%
Anesthesiology	241	0.3%	446	0.5%	558	0.5%	131.5%
Plastic surgery	184	0.2%	361	0.4%	514	0.5%	179.3%
Aesthetic plastic surgery	142	0.2%	382	0.4%	513	0.5%	261.3%
Neurology	380	0.5%	442	0.5%	476	0.5%	25.3%
Radiology	200	0.2%	294	0.3%	450	0.4%	125.0%
Rheumatology	29	0.0%	135	0.1%	194	0.2%	569.0%
Rehabilitation	77	0.1%	122	0.1%	158	0.2%	105.2%
Cardiovascular surgery	28	0.0%	46	0.0%	91	0.1%	225.0%
Allergology	52	0.1%	72	0.1%	67	0.1%	28.8%
Pathology	0	0.0%	13	0.0%	30	0.0%	n/a
Pediatric surgery	14	0.0%	38	0.0%	25	0.0%	78.6%
Emergency medicine	0	0.0%	5	0.0%	18	0.0%	n/a
Respiratory surgery	10	0.0%	15	0.0%	14	0.0%	40.0%
Residents	0	0.0%	17	0.0%	4	0.0%	n/a

The number of clinical physicians rapidly increased by 16% in the first decade, from 81,888 (34.1% of all physicians) in 1996 to 95,213 (34.3%) in 2006, while the increase in the number of clinical physicians declined by 8% in the second decade from 2006 to 2016, reaching 102,453 (32.1%) in 2016.

**Figure 1:**
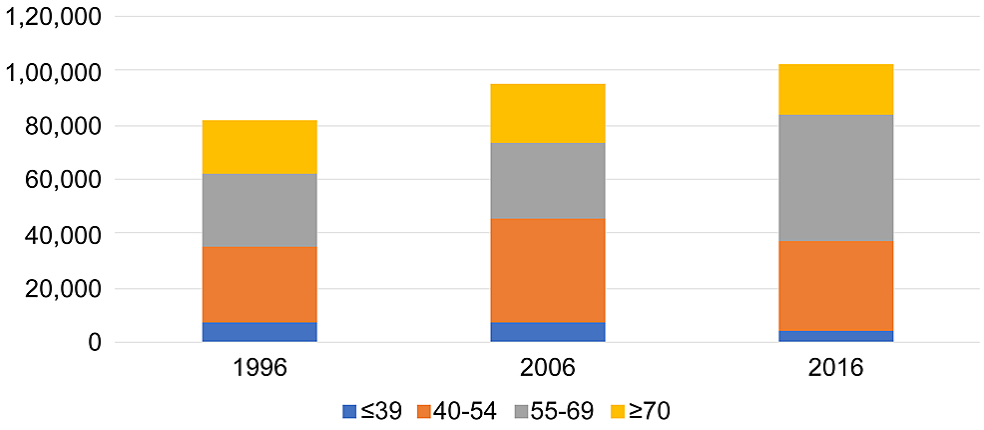
Distribution of clinical physicians by age categories in 1996, 2006, and 2016 in Japan

It should be noted that the number of clinical physicians aged 39 years or younger decreased by 35%, from 7,251 to 4,721, between 2006 and 2016, while the number of clinical physicians between the ages of 55 to 69 years greatly increased by 63%, from 28,200 to 45,984, during the same period. Thus, the aging of clinical physicians has advanced (Figure 1).

**Figure 2:**
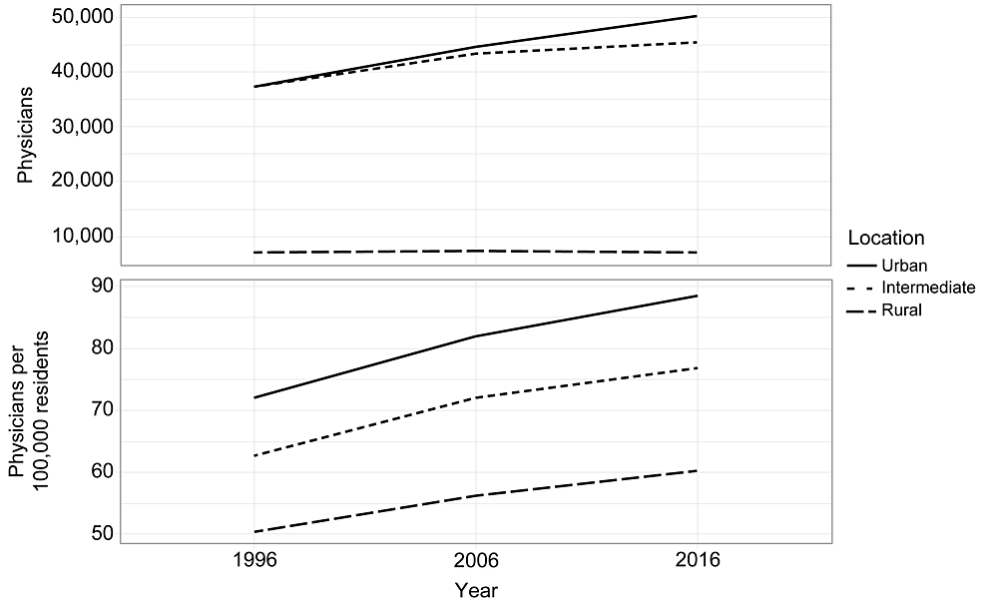
Distribution of clinical physicians by geographical area in 1996, 2006, and 2016 in Japan

The number of female clinical physicians increased by 81% between 1996 and 2016, leading to the proportion of female clinical physicians increasing from 13.1% to 18.9%. When examining the data by position, the number of clinic owners increased only by 8% during the period from 1996 to 2016, whereas that of other clinical physicians increased by 97% during the same period. In 2016, the proportion of owners among all clinical physicians decreased to 70%. When reviewing the data by specialty, the number of those specializing in primary care increased by only 8% over the period from 1996 to 2016, whereas that of clinical physicians specializing in other areas increased by 50% during the same period. Hence, the number of clinical physicians in areas other than primary care outnumbered those in primary care in 2016. Regarding the geographical criterion, between 1996 and 2016, the number of urban clinical physicians increased by 46%, whereas the number of rural clinical physicians increased by 9%; thus, the urban versus rural difference increased (Figure 2).

The multivariable logistic regression analysis illustrated in Table 2 indicates that the odds of opening clinics were significantly higher among those aged 40-54 years [reference: 25 to 39 years, odds ratio (OR): 2.12, 95% confidence interval (CI): 1.99-2.27 for the 1996-1998 cohort; OR: 2.22, 95% CI: 2.05-2.39 for the 2006-2008 cohort, and OR: 2.66, 95% CI: 2.41-2.94 for the 2014-2016 cohort] and significantly lower among females (reference: male, OR: 0.67, 95% CI: 0.60-0.75 for the 1996-1998 cohort, OR: 0.53, 95% CI: 0.47-0.59 for the 2006-2008 cohort, and OR: 0.48, 95% CI: 0.43-0.55 for the 2014-2016 cohort) and rural area practice (reference: urban, OR: 0.88, 95% CI: 0.78-0.98 for the 1996-1998 cohort, OR: 0.64, 95% CI: 0.55-0.74 for the 2006-2008 cohort, and OR: 0.76, 95% CI: 0.63-0.90 for the 2014-2016 cohort). Regarding institution, for the 1996-1998 cohort, the odds of “other hospital” was significantly higher (reference: clinic, OR: 2.16, 95% CI: 1.82-2.57). However, for the 2006-2008 and 2014-2016 cohorts, the odds were significantly lower among “academic hospital” (reference: clinic, OR: 0.82, 95% CI: 0.77-0.88 for the 2006-2008 cohort, and OR: 0.77, 95% CI: 0.71-0.84 for the 2014-2016 cohort) and “other hospital” (reference: clinic, OR: 0.88, 95% CI: 0.77-1.00 for the 2006-2008 cohort, and OR: 0.82, 95% CI: 0.69-0.98 for the 2014-2016 cohort). In addition, for the 2014-2016 cohort, “other specialty” was significantly higher (reference: primary care, OR: 1.11, 95% CI: 1.02-1.22).

**Table 2:** Logistic regression analysis of factors associated with opening clinics as an owner

1996–1998 cohort; n=181,068				2006–2008 cohort; n=192,235				2014–2016 cohort; n=246,338			
	OR	95% CI	P-value		OR	95% CI	P-value		OR	95% CI	P-value
Gender											
Male	Reference			Male	Reference			Male	Reference		
Female	0.67	0.60–0.75	<0.01	Female	0.53	0.47–0.59	<0.01	Female	0.48	0.43–0.55	<0.01
Age											
≤39 years	Reference			≤39 years	Reference			≤39 years	Reference		
40–54 years	2.12	1.99–2.27	<0.01	40–54 years	2.22	2.05–2.39	<0.01	40–54 years	2.66	2.41–2.94	<0.01
55–69 years	0.97	0.86–1.10	0.66	55–69 years	1.34	1.20–1.50	<0.01	55–69 years	1.34	1.17–1.52	<0.01
≥70 years	0.72	0.58–0.88	<0.01	≥70 years	0.93	0.78–1.13	0.48	≥70 years	0.96	0.75–1.21	0.72
Workplace											
Urban	Reference			Urban	Reference			Urban	Reference		
Intermediate	0.99	0.93–1.06	0.83	Intermediate	0.83	0.71–0.99	0.04	Intermediate	1	0.82–1.22	0.98
Rural	0.88	0.78–0.98	0.02	Rural	0.64	0.55–0.74	<0.01	Rural	0.76	0.63–0.90	<0.01
Type of institution											
Clinic	Reference			Clinic	Reference			Clinic	Reference		
Academic hospital	1.11	0.92–1.34	0.27	Academic hospital	0.82	0.77–0.88	<0.01	Academic hospital	0.77	0.71–0.84	<0.01
Other hospitals	2.16	1.82–2.57	<0.01	Other hospitals	0.88	0.77–1.00	0.05	Other hospitals	0.82	0.69–0.98	0.03
Specialty											
Primary care	Reference			Primary care	Reference			Primary care	Reference		
Others	1.06	1.00–1.14	0.05	Others	1	0.93–1.07	0.98	Others	1.11	1.02–1.22	0.02

Table 3 illustrates the specialty areas of physicians working as clinical physicians in 2016. Of 102,453 clinical physicians, 44,856 (39.1%) did not hold board-certified qualifications. In addition, 17.1% of all clinical physicians were practicing in an area outside of their board-certified qualifications. The largest proportion of clinical physicians was practicing in internal medicine (40,586 clinical physicians), of whom 23,247 (57.3%) did not hold board-certified qualifications. The proportion of physicians practicing in an area outside of their board-certified qualifications was 28.3%.

**Table 3:** Types of specialty certificates held by clinical physicians as of 2016

	Specialty	Certificate of specialty		Other certificates		No certificate	
Total	102,453	44,856	43.8%	17,521	17.1%	40,076	39.1%
Internal medicine	40,586	5,843	14.4%	11,496	28.3%	23,247	57.3%
Ophthalmology	8,395	6,446	76.8%	55	0.7%	1,894	22.6%
Orthopedics	7,796	6,066	77.8%	220	2.8%	1,510	19.4%
Pediatrics	6,581	4,898	74.4%	104	1.6%	1,579	24.0%
Otolaryngology	5,433	4,681	86.2%	37	0.7%	715	13.2%
Dermatology	5,411	3,504	64.8%	227	4.2%	1,680	31.0%
Obstetrics and gynecology	5,342	4,571	85.6%	53	1.0%	718	13.4%
General surgery	3,992	1,185	29.7%	475	11.9%	2,332	58.4%
Psychiatry	3,862	2,557	66.2%	85	2.2%	1,220	31.6%
Gastroenterology	3,389	1,435	42.3%	672	19.8%	1,282	37.8%
Cardiology	1,967	1,210	61.5%	103	5.2%	654	33.2%
Urology	1,908	1,485	77.8%	66	3.5%	357	18.7%
Neurosurgery	1,128	975	86.4%	22	2.0%	131	11.6%
Other	6,663	n/a	n/a	n/a	n/a	2,757	41.4%

## Discussion

The number of younger clinical physicians decreased dramatically from 2006 to 2016. Moreover, the number of female clinical physicians and clinical physicians aged 55 years or older increased. Apparently, clinical physicians are aging, but they tend to continue their practice even after turning 70 years old because it is relatively easy to do so; for example, they do not frequently perform invasive tests and treatments, and they see fewer patients at night. On the other hand, data in Table 1 indicate that the number of clinical physicians aged 70 years or older has declined. If this trend continues, the number of clinical physicians is likely to decrease further as more senior clinical physicians retire. According to a survey conducted by the Japan Medical Association Research Institute [13], approximately 50% of the responding clinics chose to close clinics due to a shortage of successors. It was indicated that if a succession problem were to arise in the future, it would be difficult to maintain and continue primary care in various parts of Japan [13]. Therefore, clinical physicians' aging is an important political and social issue that has yet to be resolved.

According to an estimate by the MHLW, the number of clinical physicians was expected to increase by 38% from 2004 to 2016 [6], but the actual increase was only 10% during that period, as the number of young clinical physicians decreased. There are two issues related to the decrease in the number of younger clinical physicians: firstly, few younger physicians become clinical physicians; secondly, the retention rate of younger clinical physicians is relatively low. Furthermore, it was confirmed that the rate of increase in the number of clinical physicians who own a clinic has decreased.

New factors related to physicians’ career behavior may gradually cause it to change. For example, the increase in female physicians, a general preference for a more manageable lifestyle [14,15], and other generational/cohort effects may be determined to be influential. It is possible that an increasing number of physicians are avoiding operational risks associated with opening a private clinic.

Another issue is that there has been an increase in the uneven distribution of clinical physicians. Clinics in Japan have comprehensive functions for consultation, treatment, and primary care [1]. Meanwhile, hospitals in Japan also provide healthcare services to outpatients [1]. Clinics are not necessary for a rural area as long as a local hospital can provide primary care services. Therefore, to evaluate outpatient services in an area, it may be necessary to examine both clinics and hospitals in an integrated manner.

As shown in Table 3, among all clinical physicians, 39.1% do not hold board-certified qualifications, and 17.1% practice in an area outside of their board-certified qualifications. In other words, 56.2% of all clinical physicians do not hold board-certified qualifications relevant to their area of specialty. This may be because surgeons who open a clinic change their area of surgeon specialty to internal medicine [5], and those who were working in internal medicine, such as gastrointestinal medicine and respiratory medicine, tend to open a general internal medicine clinic. Theoretically, in Japan, all licensed physicians who have completed the two-year mandatory postgraduate training can open a private clinic in any area other than anesthesiology [1].

The Japan Medical Association operates a lifelong learning program with the aim of helping physicians to effectively practice self-education [16]. A certain number of clinical physicians engage in self-education through, for example, e-learning by participating in the lifelong learning program of the Japan Medical Association. However, the participation rate in the program is unclear. It is considered that to maintain the quality of such physicians, it is ideal that the physicians continue to practice self-education through, for example, participating in a lifelong learning program and maintaining board-certified qualifications.

Limitations and future research

This study has several limitations. Firstly, the data related to the workplace environment was self-reported and, subsequently, misclassification may have occurred. Secondly, I could not acquire data to distinguish part-time clinical physicians from full-time ones. Third, this study was concerned only with the association and could not ascertain causality. The use of interviews and questionnaires could facilitate more comprehensive research. Finally, in this study, SMAs were placed in three categories based on population and population density. Thus, the results might change if the classification method were modified. Further research regarding the reasons behind the different career choices that physicians make is required, and further action must be taken to encourage young physicians to start a career as clinical physicians and address the uneven geographical distribution of clinical physicians.

## Conclusions

In recent years, the rate of increase in the number of clinical physicians in Japan has been decreasing, and the proportion of older clinical physicians has been increasing. It is necessary to continue reviewing policies on various aspects, such as securing an adequate number of clinical physicians, correcting the uneven distribution of physicians, and ensuring their quality. The major strength of the present study is that it used individual data from the national census; therefore, the sample size was large and the capture rate was high. While insufficient information on the distribution and characteristics of clinical physicians has been published, the present study provides information that may contribute to further research on the circumstances of clinical physicians and measures for securing the positions/careers of these physicians in the future.
